# Association of the triglyceride-glucose index with weight-adjusted appendicular lean mass in Chinese adolescents aged 12–18 years old

**DOI:** 10.1038/s41598-022-15012-0

**Published:** 2022-07-01

**Authors:** Jinyu Zhou, Ling Bai, Lingling Tong, Leina Jia, Wenqing Ding

**Affiliations:** 1 School of Public Health and Management, Ningxia Medical University, No. 1160, Shengli Street, Xingqing District, Yinchuan, Ningxia China; 2 Key Laboratory of Environmental Factors and Chronic Disease Control, Ningxia Medical University, No. 1160, Shengli Street, Xingqing District, Yinchuan, Ningxia China

**Keywords:** Endocrinology, Health care

## Abstract

There is no study exploring the association between triglyceride-glucose (TyG) index and skeletal muscle mass in Chinese adolescents. Therefore, the objective of this study is to explore the association between TyG index and appendicular lean mass (ALM) in Chinese adolescents. In this study, 1336 adolescents (805 boys, 60.25%) aged 12–18 years in China were randomly selected through a stratified cluster sampling. According to the tertiles of TyG index, we separated all participants into three groups, and LM was measured by Bioelectrical Impedance Analysis. The TyG index was negatively related to ALM/weight in Chinese adolescents whether stratified by gender (boys: *β* = − 0.293; girls: *β* = − 0.195; all *P* < 0.001). After adjusting for age and BMI, a significant correlation between the TyG index and ALM/weight was observed only in boys (*β* = − 0.169, *P* = 0.001). The highest TyG index tertile was significantly associated with low ALM/weight after adjusting for all covariates in the full sample (OR = 3.04, 95% CI 1.12–8.26, *P* = 0.029) and boys (OR = 4.68, 95% CI 1.22–17.95, *P* = 0.025) only in overweight/obese group. Our findings suggested elevated levels of TyG index may be a risk factor of low ALM/weight in Chinese adolescents, especially in boys.

## Introduction

Skeletal muscle mass is a vital part of body composition, and its reduction is usually considered as an age-related health problem. But recent studies indicate that the decreased muscle mass can also be found even in young people^1^. Not only does muscle mass play an important role in the consumption of energy, the absorption of glucose, and the excretion of muscle cytokines which increasing insulin sensitivity and stimulate lipolysis^2^, but also induce metabolic disorders possibly because of lower basal metabolic rate or physical activity^3^. Appendicular lean mass (ALM) or ALM adjusted by weight, height squared or body mass index (BMI) is usually used to measure skeletal muscle mass^4^. Many epidemiologic studies have also reported that skeletal muscle as an important organ for insulin disposal is closely associated with insulin resistance (IR)^5,6^. Therefore, IR might be regarded as a useful index to identify those who are vulnerable to lower muscle mass.

IR is defined as the decrease in the efficiency of insulin in promoting glucose uptake and utilization, and the prevalence of IR various from 2.2 to 10.8% in children and adolescents^7,8^. Traditionally, the primary surrogate marker to evaluate IR is homeostasis model assessment of IR (HOMA-IR)^9^. However, not only various physiological characteristics during puberty^10,11^, but the big change of basic characteristics like BMI^12^ at puberty make it hard to evaluate IR in children and adolescents. The triglyceride-glucose index (TyG index), easily calculated using fasting plasma glucose (FPG) and triglyceride (TG) levels, has been regarded as an alternative surrogate marker for appraising IR, and the accuracy of TyG index to diagnose the IR was consistent with that of HOMA-IR^14^. For large epidemiology survey, the measurement of fasting insulin is too complex to commonly measured in clinical practice, whereas FPG is measured more convenient and efficient^13^. What’s more, it was the researches that have given bright insight into how the TyG index associated with the presence of type 2 diabetes (T2DM) ^15^, cardiovascular disease^16^, strok^17^, obstructive sleep apnoea^18^, and bone health^19^ in adults. Previous studies indicated that skeletal muscle mass has an important role in IR^20^, and glucose homeostasis and lipid metabolism^21^, but study on the association between skeletal muscle mass and TyG index is limited, especially people in adolescence which is a crucial period for acquiring majority muscle mass^22^. Therefore, the objective of this study is to evaluate the association between the TyG index and weight- adjusted ALM in Chinese adolescents, and to provide a scientific basis for the prevention and treatment of lower muscle mass.

## Methods

### Study population

Through a stratified cluster sampling, we recruited a total of 1336 adolescents (805 boys, 60.25%) aged 12–18 years old at random in China from 2018 to 2020. Specifically, 11 classes from 1 Junior high School and 29 classes from 3 Senior High Schools were selected in Yinchuan city. All of them completed the questionnaire survey, anthropometric measurements, blood sample collection and clinical examination.

### Questionnaire investigation

All participants were demanded to fill in the questionnaire, which covered the basic demographic information, like the name, sex, age and birthdate.

### Anthropometrical data

Anthropometric data were collected by trained staff according to a standardized procedure. Adolescents without heavy clothes and shoes were asked to measure height, weight, and waist circumference (WC). Height was measured to the nearest 0.1 cm by an altimeter (ZH7082), and body weight was weighed within 0.1 kg with a level scale (RGT-140, Wuxi Weigher Factory Co., Ltd, China). The body mass index (BMI) was calculated by weight and height (BMI = weight (kg)/height^2^ (m^2^)). WC was also required to the nearest 0.1 cm using a no stretchable fiber measuring tape. Blood pressure was recorded after resting for 5 min in the sitting position with a sphygmomanometer, and continuously determined three times by an electronic sphygmomanometer (HEM-7012, Omron, Japan), with an interval of at least 1 min between each two measurements, and we regarded the mean value of the last two indications as blood pressure value in this study.

### Biochemical measurements

After 12 h fasting, blood samples were collected from the vein of the subjects. The levels of FPG, total cholesterol (TC), TG, high-density lipoprotein cholesterol (HDL-C), and low-density lipoprotein cholesterol (LDL-C) were measured in the laboratory by the American AU480 automatic biochemical analyzer. The TyG index was calculated by FPG and TG levels (TyG index = ln (TG (mg/dL) × FPG (mg/dL)/2)).

### Definitions

The muscle mass was estimated by weight- adjusted ALM (ALM/weight), which below < P_25_ was defined as low muscle mass in Chinese adolescents. According to the boundary points of BMI in Chinese children and adolescents^23^, we divided all participants into two groups: normal weight and overweight/obese group.

### Statistical analysis

The continuous variables were described by mean ± standard deviation (SD) or median (25–75th percentiles), and *t*-test or the Wilcoxon rank sum test of two independent samples were used for comparing the differences between genders. Multivariate liner regression analyses were used for exploring the independent associations of TyG index with ALM and ALM/weight. According to the TyG index tertiles, we separated all participants into three groups, and the differences of ALM and ALM/weight among different groups were contrasted by one-way ANOVA. Through logistic regression analysis, we further analyzed the predictive ability of TyG index to the risk of low ALM under different body weight. SPSS 26.0 was used to analyze the study results, and we treated two-side *P* value < 0.05 as being statistically significant.

### Ethics approval and consent to participate

The current study was approved by the Ethics Committee of Ningxia Medical University (2021-G053), and performed in accordance with the Declaration of Helsinki. Written informed consent was obtained from all participants and/or their legal guardians.

## Results

### Baseline characteristics of the study participants

For WC, BMI, TC, and TyG index, there was no statistic difference between both genders. But compared to boys, girls had higher DBP, TG and HDL-C (all *P* < 0.05). Meanwhile, SBP, FPG, LDL-C, ALM and ALM/weight were significantly lower in girls than in boys (all *P* < 0.05) (Table 1).

**Table 1:** Baseline characteristics of the study participants. Values are presented as means ± standard deviation, n (%) or median (interquartile ranges).

Variables	Total	Boys	Girls	*P*-value
N (%)	1336 (100)	805 (60.25)	531 (39.75)	
Age (years)	14.76 ± 1.54	14.96 ± 1.51	14.45 ± 1.55	< 0.001
WC (cm)	74.01 ± 9.98	73.78 ± 10.71	74.36 ± 8.77	0.299
BMI (kg/m^2^)	20.55 ± 3.66	20.53 ± 3.83	20.59 ± 3.39	0.747
SBP (mmHg)	111.79 ± 11.20	113.47 ± 11.48	109.25 ± 10.26	< 0.001
DBP (mmHg)	67.85 ± 8.06	66.99 ± 8.05	69.15 ± 7.90	< 0.001
FPG (mmol/L)	4.74 ± 0.66	4.77 ± 0.72	4.70 ± 0.56	0.046
TC (mmol/L)	3.95 ± 0.95	3.93 ± 0.94	3.99 ± 0.96	0.256
TG (mmol/L)	0.90 (0.71, 1.18)	0.89 (0.69, 1.16)	0.91 (0.74, 1.20)	0.018
HDL-C (mmol/L)	1.44 ± 0.38	1.41 ± 0.36	1.48 ± 0.41	0.002
LDL-C (mmol/L)	2.15 ± 0.77	2.18 ± 0.80	2.10 ± 0.73	< 0.001
ALM (kg)	19.14 ± 4.45	21.39 ± 3.97	15.72 ± 2.57	< 0.001
ALM/weight (%)	0.33 ± 0.05	0.36 ± 0.04	0.29 ± 0.03	< 0.001
TyG index	8.15 ± 0.42	8.14 ± 0.44	8.17 ± 0.39	0.112

*WC* waist circumference, *BMI* body mass index, *SBP* systolic blood pressure, *DBP* diastolic blood pressure, *FPG* fasting plasma glucose, *TC* total cholesterol, *TG* triacylglycerol, *HDL-C* high-density lipoprotein cholesterol, *LDL-C* low-density lipoprotein cholesterol, *ALM* appendicular lean mass, *TyG index* triglyceride-glucose index.

### Correlation between the TyG index and ALM or ALM/weight

Without adjustment any variable, the TyG index was negatively associated with ALM/weight whether stratified by gender (total population: *β* = − 0.226; boys: *β* =  − -0.293; girls: *β* = − 0.195; all *P* < 0.001), whereas it was positively associated with ALM only in boys (*β* = 0.117, *P* < 0.001). After adjusting for age and BMI, the association between TyG index and ALM got inverse and significant (total population: *β* = − 0.115; boys: *β* = − 0.111; girls: *β* = − 0.115; all *P* < 0.05). But compared with unadjusted variables, there was a weaker negative (total population: *β* = − 0.185; boys: *β* = − 0.169; all *P* < 0.05) or no significant (girls: *β* = − 0.117; *P* = 0.050) correlation between the TyG index and ALM/weight (Table 2).

**Table 2:** Multivariate liner regression analyses to evaluate the independent associations of TyG index with ALM and ALM/weight.

	Total			Boys			Girls		
	β	SE	P	β	SE	P	β	SE	P
**Unadjusted**									
ALM	0.050	0.003	0.070	0.117	0.004	0.001	0.051	0.007	0.243
ALM/weight	− 0.226	0.224	< 0.001	− 0.293	0.346	< 0.001	− 0.195	0.593	< 0.001
**Model 1**									
ALM	− 0.115	0.004	0.006	− 0.111	0.005	< 0.001	− 0.115	0.008	0.034
ALM/weight	− 0.185	0.426	< 0.001	− 0.169	0.534	0.001	− 0.117	0.833	0.050

The Model 1 is adjusted for age, BMI. *ALM* appendicular lean mass, *TyG index* triglyceride-glucose index.

### Comparation of ALM and ALM/weight according to the TyG index tertile

We compared the means of ALM and ALM/weight according to the TyG index tertile within the full sample and stratified by gender in Table 3. A decreased trend can be clearly observed by the increasing TyG index tertiles in terms of ALM/weight in the full samples and both genders (all *P* < 0.001), although not all post hoc pairwise comparisons achieved statistical significance (*P* < 0.05). However, the levels of ALM did not differ among the TyG index tertiles in the full samples and both genders (all *P* > 0.05).

**Table 3:** Comparing means of ALM and ALM/weight according to the TyG index tertile.

	TyG index			*P*-Value	Pairwise comparisons		
	T1	T2	T3		T1–T2	T1–T3	T2–T3
**Total**							
ALM	19.05 ± 4.19	18.83 ± 4.53	19.52 ± 4.61	0.063	0.460	0.117	0.021
ALM/weight	0.34 ± 0.05	0.33 ± 0.05	0.32 ± 0.05	< 0.001	0.001	< 0.001	< 0.001
**Boys**							
ALM	21.01 ± 3.72	21.36 ± 4.08	21.81 ± 4.11	0.054	0.303	0.016	0.188
ALM/weight	0.37 ± 0.04	0.36 ± 0.04	0.34 ± 0.05	< 0.001	0.039	< 0.001	< 0.001
**Girls**							
ALM	15.61 ± 2.35	15.58 ± 2.61	15.98 ± 2.57	0.274	0.903	0.196	0.140
ALM/weight	0.30 ± 0.03	0.29 ± 0.03	0.28 ± 0.03	0.001	0.315	< 0.001	0.004

*ALM* appendicular lean mass, *TyG index* triglyceride-glucose index.

### ALM/weight according to TyG index and BMI

**Figure 1:**
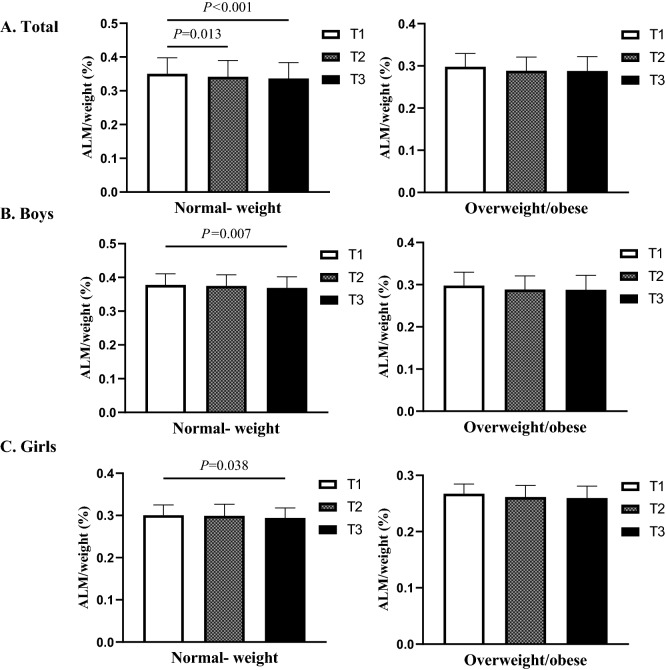
ALM/weight in total population (**A**), boys (**B**) and girls (**C**) according to the TyG index tertile in the normal- weight and overweight/obese groups. *ALM* appendicular lean mass, *TyG index* triglyceride-glucose index**.**

In Fig. 1, we divided all participants into two group, normal- weight and overweight/obese to further analyze the relationship between TyG index and ALM/weight. In normal- weight group, adolescents with the highest TyG index tertile tended to have the lowest ALM/weight in the full samples (*P* = 0.001) and boys (*P* = 0.024), but not in girls (*P* = 0.093). In the overweight/obese group there was significant difference in terms of ALM/weight among different TyG index tertile only in boys (*P* = 0.012).

### Association between TyG index and low ALM/weight in different weight group

The power of the TyG index to predict the risk of low ALM/weight were shown in Table 4. In normal- weight group, no significant association was observed between TyG index tertiles and low ALM/weight (all *P* > 0.05). Whereas in overweight/obese, the significant association between highest TyG index tertile and low ALM/weight was identified after adjusting for all covariates in the full sample (OR = 3.04, 95% CI 1.12–8.26, *P* = 0.029) and boys (OR = 4.68, 95% CI 1.22–17.95, *P* = 0.025); however, this was not observed in girls (*P* = 0.948).

**Table 4:** Logistic regression analysis of TyG index tertile and low ALM under different body weight States with adjustment for age.

TyG index	Total		Boys		Girls	
	ORs (95%CIs)	*P*	ORs (95%CIs)	*P*	ORs (95%CIs)	*P*
**Normal weight**						
T1	1		1		1	
T2	0.94 (0.59, 1.50)	0.801	0.77 (0.23, 2.56)	0.667	1.06 (0.63, 1.78)	0.822
T3	1.20 (0.74, 1.94)	0.459	0.60 (0.15, 2.52)	0.489	1.42 (0.83, 2.43)	0.196
**Overweight/obese**						
T1	1		1		1	
T2	2.25 (0.75, 6.73)	0.149	2.62 (0.59, 11.68)	0.208	2.93 (0.23, 36.94)	0.405
T3	3.04 (1.12, 8.26)	0.029	4.68 (1.22, 17.95)	0.025	1.07 (0.16, 7.09)	0.948

*ALM* appendicular lean mass, *TyG index* triglyceride-glucose index.

## Discussion

This current study indicated that there was a negative relationship between the TyG index and ALM/weight in Chinese adolescents of both genders. After adjusting for age and BMI, our observation on the correlation between the TyG index and ALM/weight was significant only in boys. Furthermore, a decreased trend can be clearly observed by the increasing TyG index tertiles in terms of ALM/weight in the full samples and both genders. Moreover, the significant association between highest TyG index tertile and low ALM/weight was identified in the full sample and boys in overweight/obese group after adjusting for all covariates.

IR not just causes the occurrence of diabetes, but also is an important risk factor for low muscle mass^24,25^. Euglycemic hyperinsulinemia clamp has been verified to be the gold standard methods for appraising IR^26^, but it is its disadvantages like time consuming, high price, and complex operation that make it unappropriated to apply in large epidemiological studies and clinical examination. Not only TG, but also FPG plays a vital role in muscle mass and muscle IR^27^, and the TyG index is a better surrogate because it can well combined TG and FPG. In addition, study has showed that the TyG index is superior to HOMA-IR in predicting cardiometabolic disease such as metabolic syndrome^28^, arterial stiffness^29^, and T2DM^30^, which are closely associated with low muscle mass. Therefore, compared with the gold standard and HOMA-IR, the TyG index might be more suitable for assessing IR large epidemiology survey.

Our results demonstrated that the IR was positively related to ALM, but this positive correlation would get inverse after using the weight-adjusted ALM. Consistent with our study, a Chinese twin study^31^ found a positive relation between lean body mass and IR evaluated by HOMA-IR, whereas data from a Korean National Health and Nutritional Examination Survey IV and V (KNHANES)^32^ and a national observational study conducted in Chinese school-age children^33^ reported that with adjustment of age, the TyG index was negatively associated with LM. Animal studies also indicated that there was a significantly negative association between muscle mass and the risk of diabetes, and it is possible that the proper increase of muscle mass could reduce the risk of obesity and IR in mice^34,35^. The inverse association may be related to the compositions of body weight (fat mass and LM): the higher the proportion of LM, the lower the proportion of fat mass. Therefore, it cannot be ruled out that this association is caused by low fat mass rather than high LM. What’s more, a prospective cohort study demonstrated that IR independently influenced the LM in adults without diabetes^36^. The balance between synthesis and degradation of muscle protein can be disrupted by the elevated IR through some biological pathways^37^. Muscle mass reduction can also induce IR through lowering insulin mediated glucose absorption^38^.

In our study, the increase risk of low ALM/weight was significantly associated with the highest TyG index tertile after adjusting for all covariates only in overweight/obese boys. It seemed that there are sex discrepancies in the association between low ALM/weight and IR, which can be explained by the differences of fat distribution and different levels of insulin sensitivity in the adipose tissue. It is well known that the body fat in women is mainly accumulated in the hips and thighs, whereas in men is distributed in the trunk and abdominal visceral fat, which have been found to be related to IR^39^. Moreover, a previous animal research revealed that the insulin sensitivity of adipocytes in male was lower than that of female ones^40^. Inflammation associated with low ALM also plays a major role in IR. The higher levels of IL-6 in overweight/obese individuals with low ALM could active the pathways related to IR, including transcription nuclear factor-kB and JUN N-terminal kinase pathways^41,42^.

There are some limitations worthy of our attention. First, this study is an observational study, which made it not appropriate to assess the causality between the TyG index and ALM. Second, although we adjusted for age, and BMI, other confounding factors like physical activity and lifestyles were not considered in the present study. Third, the participants of our study were only the Chinese adolescents aged 12–18, so the results may not be applicable to other populations due to the racial difference of triglyceride levels. Furthermore, data pertaining to muscle strength and physical performance are limited in our study. Finally, there is no comparation among the TyG index and other methods evaluating IR, such as HOMA-IR, and quantitative insulin sensitivity check index (QUICKI) in this study. Nevertheless, this is the first study to assess the association between IR estimated by the TyG index and weight- adjusted ALM in people in adolescence, which is a crucial period for acquiring majority muscle mass. Besides, compared with previous studies, we further explore the association between TyG index and weight- adjusted ALM in the different weight status, which may provide new insights in the association between IR and muscle mass in adolescents who have lower muscle mass but overweight/obese.

In conclusion, elevated TyG index may be a risk factor of low ALM/weight in Chinese adolescents, especially in boys. The TyG index might be an alternative surrogate to identify the high-risk group of low muscle mass. Therefore, further studies are still needed to evaluate the efficacy of TyG in predicting adverse clinical outcomes of sarcopenia and cardiometabolic diseases.

## Data Availability

The datasets used and/or analyzed during the current study are not publicly available but are available from the corresponding author on reasonable request.
